# JAK2 inhibition has different therapeutic effects according to myeloproliferative neoplasm development in mice

**DOI:** 10.1111/jcmm.12608

**Published:** 2015-07-14

**Authors:** Franck Debeurme, Catherine Lacout, Claudine Moratal, Rebecca G Bagley, William Vainchenker, Francisco Adrian, Jean-Luc Villeval

**Affiliations:** a Inserm, U.1009, Institut Gustave Roussy (IGR), Université Paris XIVillejuif, France; b iBV, CNRS UMR7277, INSERM U1091, Université Nice-Sophia AntipolisNice, France; c Sanofi OncologyCambridge, MA, USA

**Keywords:** JAK2 inhibitor, myeloproliferative neoplasms, fibrosis, preclinical murine models

## Abstract

JAK2 inhibition therapy is used to treat patients suffering from myeloproliferative neoplasms (MPN). Conflicting data on this therapy are reported possibly linked to the types of inhibitors or disease type. Therefore, we decided to compare in mice the effect of a JAK2 inhibitor, Fedratinib, in MPN models of increasing severity: polycythemia vera (PV), post-PV myelofibrosis (PPMF) and rapid post-essential thrombocythemia MF (PTMF). The models were generated through JAK2 activation by the JAK2^V617F^ mutation or MPL constant stimulation. JAK2 inhibition induced a correction of splenomegaly, leucocytosis and microcytosis in all three MPN models. However, the effects on fibrosis, osteosclerosis, granulocytosis, erythropoiesis or platelet counts varied according to the disease severity stage. Strikingly, complete blockade of fibrosis and osteosclerosis was observed in the PPMF model, linked to correction of MK hyper/dysplasia, but not in the PTMF model, suggesting that MF development may also become JAK2-independent. Interestingly, we originally found a decreased in the JAK2^V617F^ allele burden in progenitor cells from the spleen but not in other cell types. Overall, this study shows that JAK2 inhibition has different effects according to disease phenotypes and can (*i*) normalize platelet counts, (*ii*) prevent the development of marrow fibrosis/osteosclerosis at an early stage and (*iii*) reduce splenomegaly through blockage of stem cell mobilization in the spleen.

## Introduction

Classical BCR-ABL-negative myeloproliferative neoplasms (MPN) include polycythemia vera (PV), essential thrombocytemia (ET) and primary myelofibrosis (PMF). They are malignant hemopathies resulting from the transformation of a multipotent haematopoietic stem cell (HSC). The common mechanism of transformation is the constitutive activation of the cytokine receptor/JAK2 pathway that leads to the myeloproliferation. The acquired point mutation JAK2^V617F^ is the most prevalent (95% of patients with PV and 60% of patients with ET or PMF) 1. JAK2^V617F^ is a gain-of-function mutation leading to activation of cytokine receptor pathway, such as the erythropoietin or the thrombopoietin (MPL) receptors. Activating MPL^W515X^ mutations are also observed in less than 10% PMF 2. In a minority of MPN, loss-of-function mutations are also observed for two negative regulators of the JAK/STAT pathways LNK and CBL 3,4. Very recently, whole-exome sequencing identified recurrent somatic mutations of calreticulin (CALR) 5,6. These mutations are found in ET and PMF patients devoid of JAK2 or MPL mutations but not in PV. Mutant CALR induces JAK2/STAT activation by a yet unknown mechanism and patients with CALR mutations respond to JAK2 inhibitors 7. In addition to these driver or phenotypic mutations activating the JAK/STAT pathway, a growing list of somatic mutations in genes that are commonly mutated in other haematological myeloid malignancies have been reported in patients with MPNs at frequencies from 1% to 25% for each of them. They affect genes involved in splicing, epigenetic regulation, or tumour suppressors that may have dual roles in both disease initiation and progression 8.

The discovery of JAK2^V617F^ has rapidly led to the development of JAK2 inhibitors with different kinase specificities and biological/clinical activities. Ruxolitinib has been approved for the treatment of myelofibrosis inducing major effects on splenomegaly, constitutional symptoms related to inflammation and quality of life but minor effects on survival and the JAK2^V617F^ clones. It has been recently tested in PV patients resistant to hydroxyurea offering a new option for the treatment of PV 9. Fedratinib (also called SAR302503/TG101348) displays a higher degree of kinase selectivity for JAK2 *versus* the other JAK family members than ruxolitinib or other JAK2 inhibitors 10. This small molecule has also shown efficacy in treating PMF patients with reduction in splenomegaly and normalization of blood counts 11. It has been assessed in JAK2^V617F^ retrovirally transduced mice and KI mice 12,13. In these human PV-like mouse models, Fedratinib showed a reduction in white blood cells (WBC), spleen size, histological defects and erythroid dysplasia including tissue progenitor/precursors and haematocrit. An effect on allele burden was observed in the retroviral (RV) model, but no effect on disease-initiating cells in a KI model. Effect on platelets or fibrosis was not evaluated in these models that did not develop very abnormal levels of platelets or fibrosis 12–15.

In this study, we decided to test anti-JAK2 therapeutic efficacy, using Fedratinib, in three different murine MPN models: PV, post-PV MF (PPMF) and post-ET MF (PTMF). While some parameters, as splenomegaly, leucocytosis and erythroid hyperplasia varied in a similar way in all models, some responses involving platelets, granulocytes, fibrosis or osteosclerosis varied according to disease models and severity. JAK2 inhibition decreases the JAK2^V617F^ allele burden in progenitor cells from the spleen but not in mature cells or marrow progenitor cells. Overall, this study describes three preclinical models of MPN, recapitulates changes induced by a JAK2 inhibition and finally suggests that it could (*i*) prevent the development of fibrosis if applied in early-phases, (*ii*) normalize platelet count and (*iii*) reduce splenomegaly through reduction in stem cell mobilization from the marrow to the spleen.

## Materials and methods

### Generation of PV, PPMF and PTMF models

**Figure 1:**
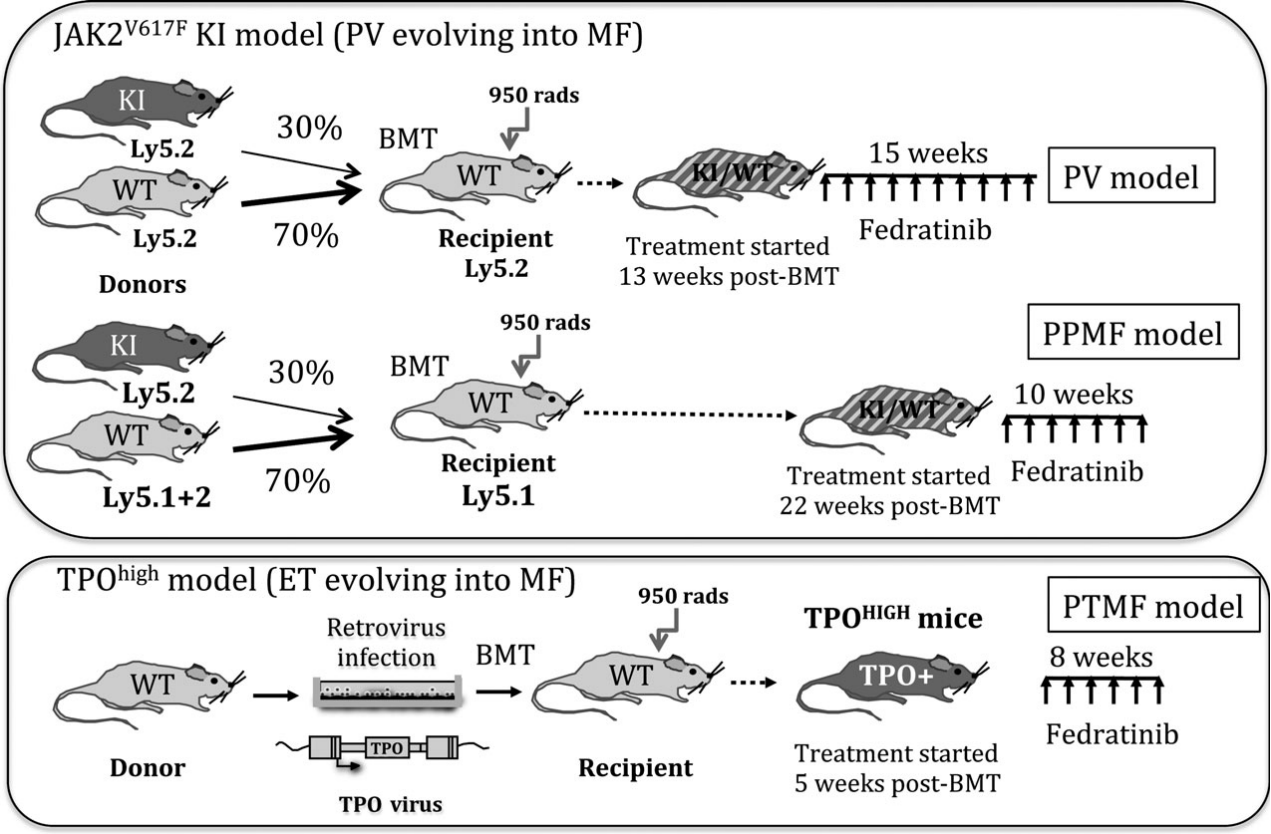
Myeloproliferative neoplasms (MPN) animal models developed to test the therapeutic utility of Fedratinib. We developed three models of MPN corresponding to three degrees of disease severity. The polycythemia vera (PV) model is the milder one but it slowly evolves into post-PV myelofibrosis (PPMF), a more severe form of MPN with fibrosis, reduction in polycythemia and possible anaemia. The post-essential thrombocythemia MF (PTMF) form is the most severe form of MPN starting with initial thrombocytosis, leucocytosis and anaemia and progressively evolving into severe pancytopenia and premature death. The PV or PPMF murine models were derived from lethally irradiated recipient mice (9.5 Gy) transplanted with a mixture of BM cells (BMT) collected from JAK2^V617F^ KI (1/3) and WT (2/3) mice 16. These mice develop a disease mimicking human PV evolving into severe PPMF around 7 months after transplantation 16 and were studied from 13 to 28 weeks after transplantation for the PV phenotype or from 22 to 32 weeks after transplantation for the PPMF phenotype. To monitor the response of neoplastic cells (also called JAK2^V617F^ allele burden) to the treatment, in the PPMF model, we transplanted a mixture of Ly5.1+2 WT cells and Ly5.2 JAK2^V617F^ KI cells into Ly5.1 WT recipient mice. JAK2^V617F^ allele burden was measured by monitoring the Ly5.2 allele by FACS analysis. Competitive WT cells and residual endogenous reconstitution from the WT recipient were measured using the Ly5.1+2 alleles or the Ly5.1 allele respectively. The PTMF model (called TPO^high^) derives from the recipient mice transplanted with BM cells transduced with a retrovirus (RV) expressing the TPO gene. Severe PTMF quickly occurred around 3 months after transplantation 18. Briefly, 4 days after 5-fluorouracil (5-FU) treatment (150 mg/kg), BM cells from two WT C57Bl/6 femurs were co-cultivated for 4 days with 10^5^ MPZenTPO virus-producing GP/E-86 cells in 20 mL DMEM containing IL3, SCF and 20% FCS. Non-adherent cells were removed and injected into lethally irradiated congenic recipient mice. Mice were treated with Fedratinib as described in materials and methods by oral gavage Semel in Die (SID).

The conditional flexed JAK2 KI mice have been previously described 16. To express the mutation, KI mice were crossed with VavCre transgenic (TG) mice 16,17. These heterozygous recombined JAK2^V617F/WT^ mice with a wild type (WT) and a mutated *Jak2* allele, termed as JAK2^V617F^ KI mice, were used to generate the PV or PPMF models (Fig.1). The previously described TPO^high^ mice 18 were used to generate the PTMF model (see Fig.1 for details).

### Treatment and analysis of mice

The Fedratinib powder was diluted in water containing 0.5% methylcellulose and 0.05% Tween 80. Solutions were administrated once a day (SID) at escalading doses by oral gavage. Maximum tolerated doses (MTD) decreased as disease severity increased and were evaluated, according to high mortality, at 150, 190 or 240 mg/kg (mpk) for the PTMF, PPMF or PV model respectively. Therefore, in the PV model, mice were treated for 15 weeks from 60 to 190 mpk (190 mpk for 8 weeks). In the PPMF model, mice were treated for around 10 weeks from 100 to 150 mpk (150 mpk for 4 weeks). In the PTMF model, mice were treated for 8 weeks from 60 to 120 mpk (120 mpk for 3 weeks) (Fig.1).

Haemoglobin (Hb), mean corpuscular or globular volume (MCV/MGV), haematocrit, red blood cell (RBC), platelet and WBC counts were determined using an automated counter (MS9; Schloessing Melet, Osny, France) on blood collected from the retro-orbital plexus in citrated tubes. BM cells were removed by flushing both femurs. Spleens were weighted and single cell suspensions were prepared. Histology and flow cytometry of blood, BM and spleen were used as described in supplemental data.

### Progenitor cell study

Progenitor cell assays were carried out in 1 ml methylcellulose ‘MethoCult 32/34’ (Stemcell Technologies, Vancouver, Canada) without stimulus or maximally stimulated by interleukin 3 (IL3), IL6, TPO, SCF and EPO. Cultures in duplicate were scored after 2 days for colony-forming unit-erythroid (CFU-E) assays and 8 days for burst forming unit-erythroid (BFU-E) and CFU-granulocyte macrophage (CFU-GM) assays. Total progenitor cell number was calculated assuming that one femur represents 6% of the total marrow and from the total number of cells isolated from the spleen.

### Statistical analysis

Results are presented as mean ± SEM and data were analysed with the two-tailed Student's *t*-test.

## Results

### Fedratinib treatment, pharmacokinetic (PK) and pharmacodynamic (PD) analysis

To test the therapeutic utility and efficacy of Fedratinib in classical MPN, we treated mice developing diseases mimicking human PV, PPMF and PTMF (Fig.1). Mice were treated as described in materials and methods for PD/PK analysis and determination of MTD. PK studies revealed in the PV model that a single dose of 120 or 240 mpk was able to maintain a plasma concentration above the IC_50_ for 18 or >24 hrs respectively. IC_50_ (190 nM) was determined, using the Phosphoflow assay, as the dose was able to decrease by half the level of P-Stat3 (or P-Stat5) induced by TPO in Baf3MPL cells. Using Western blot analysis as a PD assay, a dose of 190 mpk was able to completely suppress Stat3 and Stat5 phosphorylation observed in BM cells after a 10 min. Epo (10 U/ml) and IL3 (0.1 μg/ml) stimulation (data not shown).

### Effect on blood parameters

**Figure 2:**
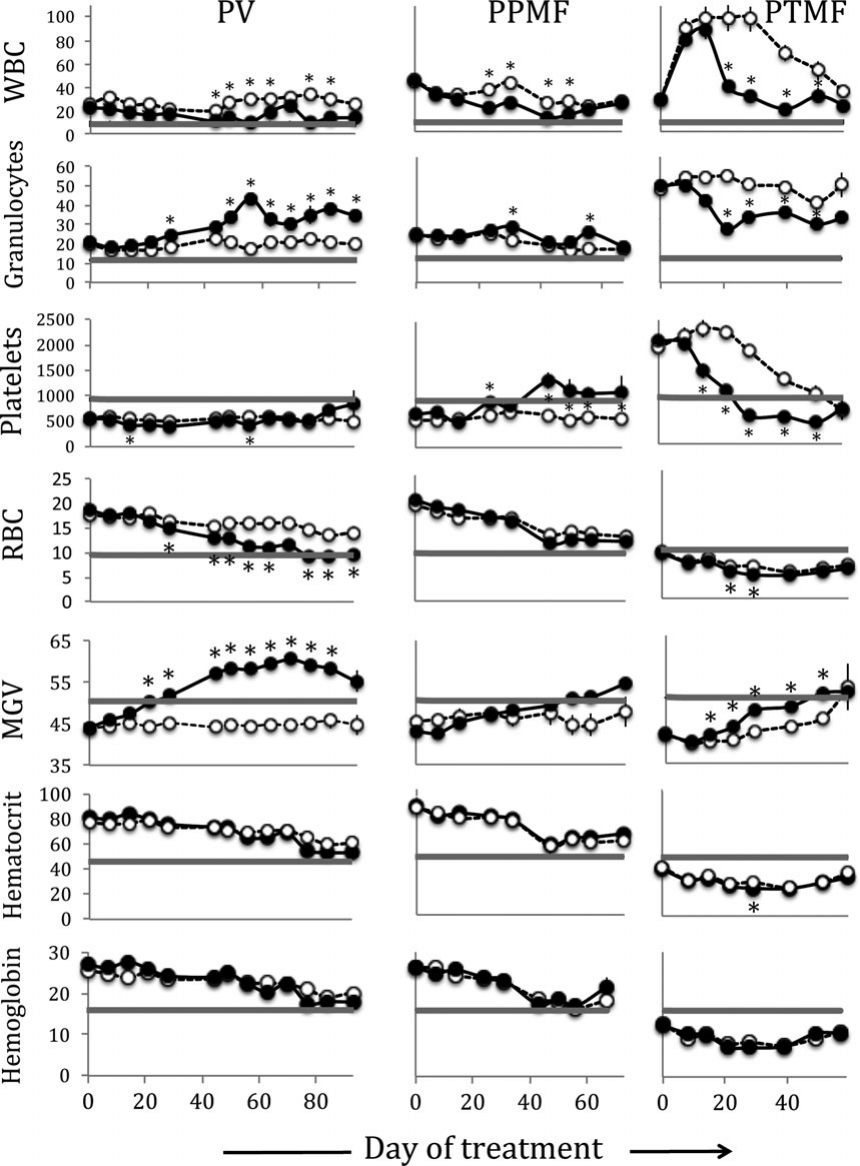
Effect of Fedratinib on blood cell parameters in polycythemia vera (PV), post-PV myelofibrosis (PPMF) and rapid post-essential thrombocythemia MF (PTMF) mice. Blood parameters from Fedratinib (filled circles, *n* = 3–15)- and vehicle (open circles, *n* = 3–15)-treated mice were studied. WBC: white blood cells (10^3^ cells/μl), granulocytes (%), platelets (10^3^/μl), RBC: red blood cells (10^6^ cells/μl), MGV: mean globular volume (or mean corpuscular volume, MCV) (fl) and haematocrit (%) and haemoglobin (g/dl). The grey lines indicate the normal values measured in WT C57/Bl6 mice. Results are mean values ± SEM, *P ≤ 0.05.

In all MPN models, Fedratinib induced a reduction in WBC counts towards normal values after 20 days of treatment (Fig.2). This was clearly observed for the high leucocytosis of PTMF mice, where a decrease from 100 × 10^9^ WBC/l to 30 × 10^9^ WBC/l was observed after 1 month of treatment. The drop was mainly in the PTMF model because of a decrease in Gr1^+^/CD11b^+^ (from 26 ± 3% to 6 ± 1% after 20 days of treatment, *P* < 0.01). In contrast, in the PV and PPMF models, the percentage of Gr1^+^/CD11b^+^ or granulocytes increased, especially in the PV model (Fig.2), and the modest drop in WBC was mainly because of a decrease in CD4^+^ cells (maximal drops from 35 ± 1 to 17 ± 2% at 63 days or from 19 ± 1 to 13 ± 3% at 31 days in the PV or PPMF models, respectively, *P* < 0.05). This difference maybe because of the type of circulating myeloid cells, immature (myelemia) in the TPO^high^ model 18 and mature in the JAK2^V617F^ KI models.

Platelet level responses to Fedratinib also varied according to the models (Fig.2). In the PTMF model, thrombocytosis was initially observed (ET-like phenotype) but dropped concomitantly to the development of myelofibrosis (see vehicle). Fedratinib suppressed thrombocytosis after 3 weeks of treatment, but interestingly did not worsen thrombopenia observed with the vehicle-treated mice around 50 days after treatment (around 0.5 × 10^6^ platelets/μl). In the PV and PPMF models, a mild thrombopenia was observed and instead of worsening the phenotype, Fedratinib treatment normalized platelet counts. In the PPMF model, a durable increase was observed from 43 to 63 days (end-point) of treatment (*P* < 0.01). In the PV model, the same tendency towards a platelet level increase was observed after 93 days of treatment.

Strikingly, the high level of RBC in the PV model dropped under Fedratinib treatment to progressively reach normal levels (Fig.2). In the PPMF model, RBC numbers drop during the survey because of MF development and Fedratinib did not modify this drop. In the PTMF model, the very low RBC levels only slightly worsened during Fedratinib treatment. The MGV clearly increased in response to Fedratinib (Fig.2) in all models. From the initial microcytosis because of iron deficiency in JAK2^V617F^ KI mice 19, animals even developed a macrocytosis for the PV model and reached normal MGV in both MF models. As a consequence, haematocrit and Hb levels (Fig.2) were hardly modified by the treatment. Reticulocyte counts decreased in the PV (day 85) and PPMF (day 67) models from around 8–10% to around 3–4% (normal values around 3%) showing restraining of erythropoiesis in the JAK2^V617F^ models. In contrast, in the PTMF model, the values remained close to 10% for the Fedratinib- and vehicle-treated mice showing that Fedratinib did not worsen stressed erythropoiesis in high oxygen demand.

### Effect on BM and spleen

**Figure 3:**
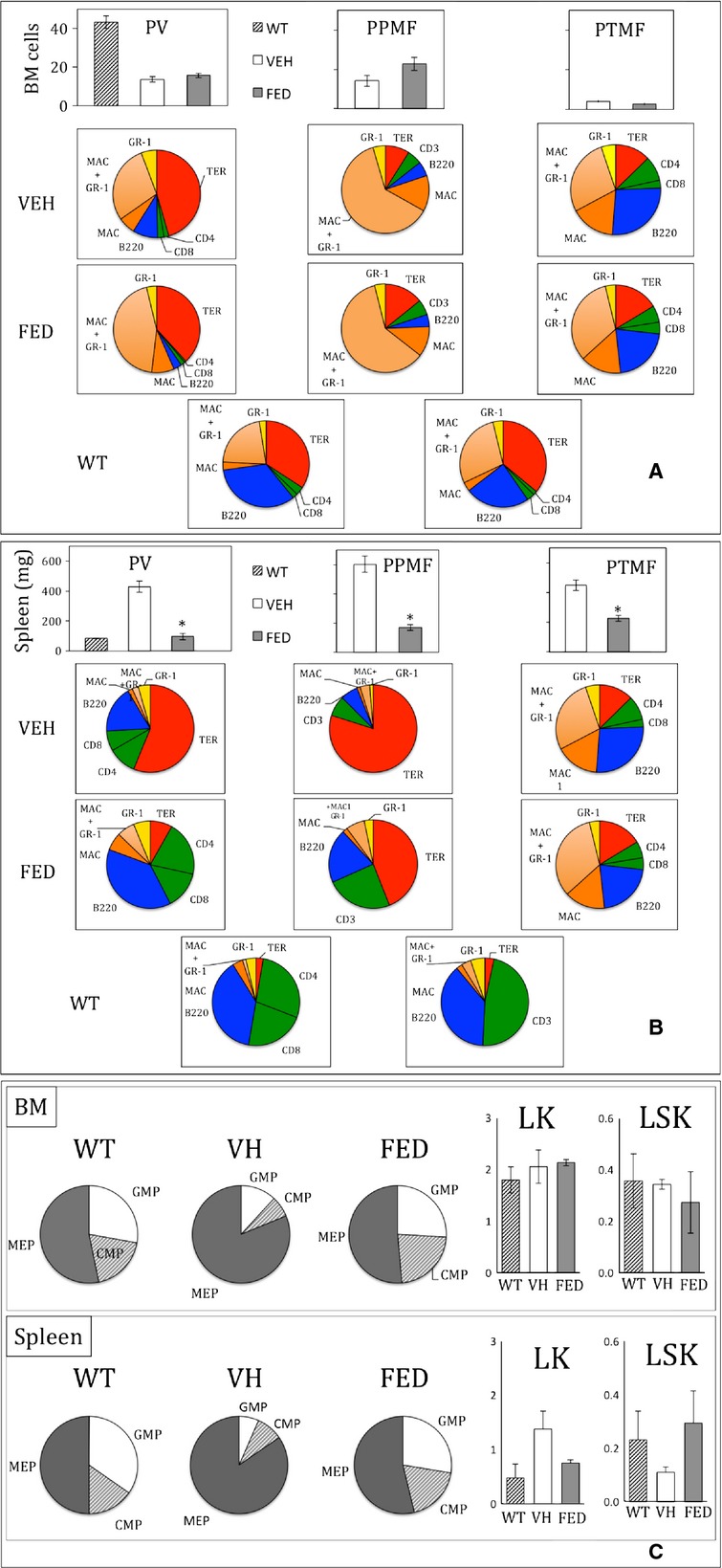
Effect of Fedratinib on BM and spleen cell composition of polycythemia vera (PV), post-PV myelofibrosis (PPMF) and rapid post-essential thrombocythemia MF (PTMF) mice. (**A**) BM cell numbers per femur (×10^6^) (top) and cell type percentages in vehicle (VEH, top, white column)-, Fedratinib (FED, middle, grey column)-treated mice (*n* = 6–7) and normal WT C57/Bl6 mice (WT, bottom, striped column) (*n* = 4). Cell types were determined by FACS analysis with antibodies directed against Gr-1, CD11b (Mac1), Terr119/CD71 (TER), CD3 (or CD4 and CD8) and B220. (**B**) Spleen weight and cell type percentages in vehicle (VEH, top)-, Fedratinib (FED, middle)-treated mice (*n* = 6–7) and normal WT C57/Bl6 mice (WT, bottom) (*n* = 4). Cell types were determined by FACS analysis as in **A**. Normal spleen cell composition was restored by Fedratinib in the PV model. (**C**) Progenitor cell per cent in BM and spleen from WT C57/Bl6 mice (WT, bottom) (*n* = 3) and from mice developing PPMF and treated by Fedratinib (FED) or vehicle (VH) (*n* = 4). Lin^−^ cell subpopulations (Gr1, CD11b, B220, CD3 and Ter119 negative) were identified according by FACS analysis: LSK (progenitor cells with multipotent activity, lin^−^, c-kit^+^, Sca-1^+^) and subpopulation of LK myeloid progenitor cells (Lin^−^, c-kit^+^) including MEP (megakaryocyte-erythroid progenitors, LK, CD34^−^, FcRgII/III^−^), GMP (granulocyte-macrophage progenitors, LK, CD34^+^, FcRgII/III high) and CMP (common myeloid progenitors, LK, CD34^+^, FcRgII/III low). Fedratinib restored normal proportions of MEP, GMP and CMP in BM and Spleen. All experiments were carried out at the end of treatment, after around 8, 10 or 15 weeks of treatment in the PTMF, PPMF or PV models respectively. Results represented the percent of cell types per singlet and are mean values ± SEM, *P ≤ 0.05.

BM cellularity was low (Fig.3A) in all models, particularly in the PTMF model, and Fedratinib treatment did not increase it. Similarly, Fedratinib did not modify BM cell type composition. Compared to WT mice, the percentage of myeloid cells (Gr-1/Mac1 and Mac1) in the PV and PPMF models remained high while B cells were very low. In the PTMF model, the percentage of myeloid (especially Mac1^+^) cells remained high and those of erythroblasts remained low.

Splenomegaly was observed in all models, particularly in the PPMF model (Fig.3B). It was mainly because of erythroid hyperplasia in the PV/PPMF models and of myeloid hyperplasia in the PTMF model. Fedratinib sharply decreased spleen weight to almost normal values in all models. In the PV model, spleen cell composition almost returned to normal values with a large decrease in erythroblasts (Ter-119^+^/CD71^+^) cells and an increase in the percentages of B and T cells. The same tendency was observed in the PPMF model but the level of erythroblasts still remained elevated. In very sharp contrast, the spleen cell composition in the PTMF model was unchanged by the treatment.

Fedratinib had a profound effect on megakaryocyte (MK) hyperplasia except in the PTMF model. In the PV model, Fedratinib induced a potent decrease in MK density in the spleen but not in BM (Fig. S1A and B). Preliminary results also showed a reduction after treatment of the high ploidy observed in JAK2^V617F^ KI mice (mean ploidy from 20 ± 2N before to 14 ± 1N after treatment, WT mice 16 ± 2N, *n* = 4). In the PPMF model, Fedratinib induced a decrease in MK density and size in spleen. In BM, only MK size was reduced (Fig. S1C). Total transforming growth factor (TGF)-β1, a MK-derived cytokine involved in fibrosis, tended to decrease in BM and spleen (Fig. S2). In the PTMF model, no effect in spleen and BM MK was observed, probably because of the extreme MK hyperplasia observed in the TPO^high^ model (Fig. S1A and B).

At the progenitor cell level, methylcellulose cultures revealed a decrease in the total high numbers of spleen CFU-GM, BFU-E and CFU-E in all models (*P* < 0.05) after Fedratinib treatment. In the BM, there was a decrease in the total CFU-E number only for the PV model and an increase in the total BFU-E number for the PPMF model, all other total number of BM progenitor cells showing no variation during treatment. In the PPMF model, flow cytometry analysis (Fig.3D) showed that Fedratinib strikingly restored normal proportions of BM and spleen MEP, GMP, CMP and overall total progenitor cells (LK/LSK cells) suggesting that defects in immature cells were corrected in contrast to, or before, progenies.

### Histology and Fibrosis of the BM and spleen

**Figure 4:**
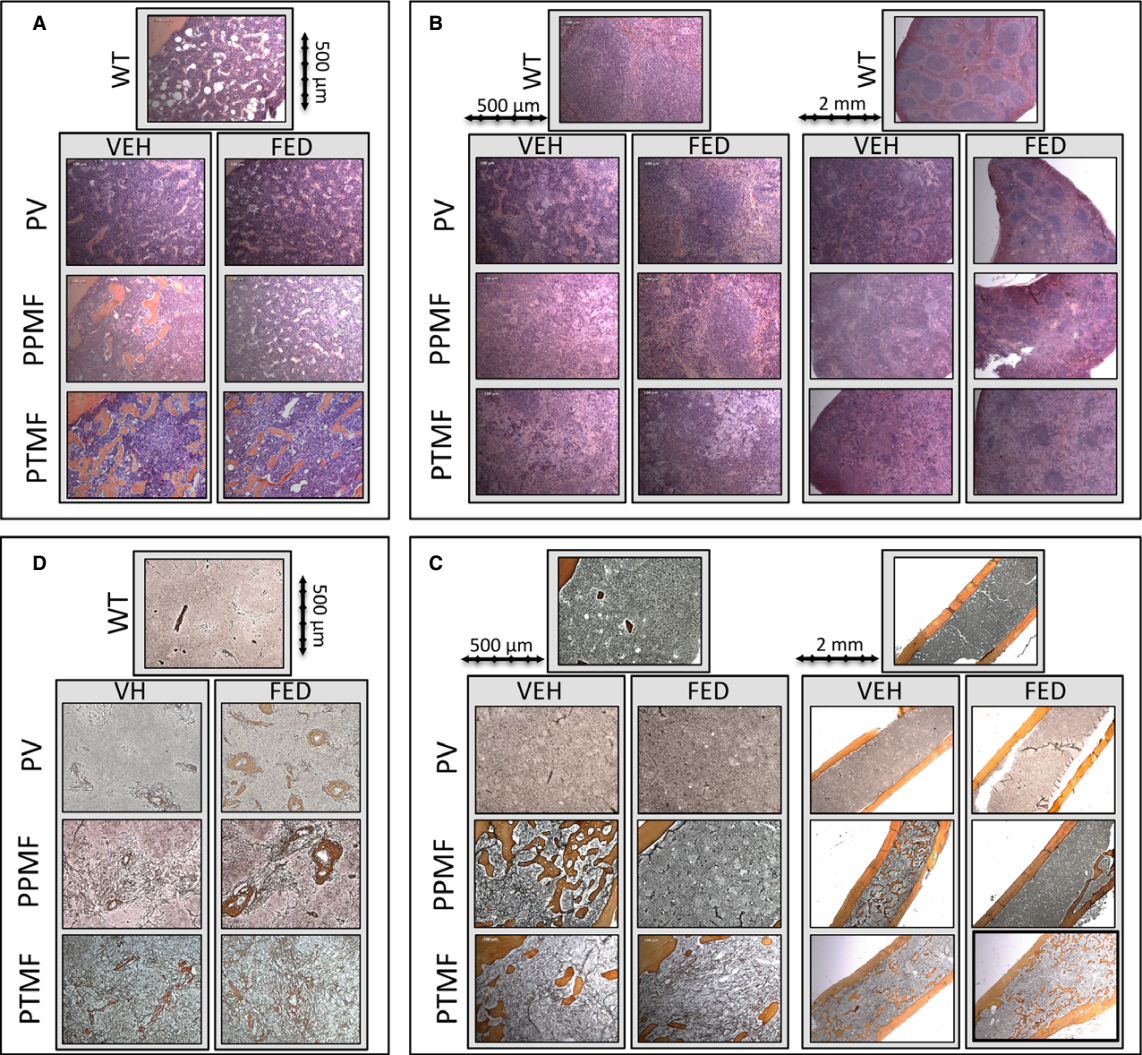
Histology of WT mice and polycythemia vera (PV), post-PV myelofibrosis (PPMF) and rapid post-essential thrombocythemia MF (PTMF) mice treated by Fedratinib or vehicle. BM and spleen were stained with haematoxylin and eosin/safran (HES) (**A** and **B**) and silver (**C** and **D**). (**A**) BM HES staining 10× lens. BM osteosclerosis is shown in vehicle-treated PPMF and PTMF mice. Fedratinib-treated PPMF mice do not display osteosclerosis in contrast to Fedratinib-treated PTMF mice. High density of fibrotic tissues responsible for the low cellularity is shown in BM PTMF mice. (**B**) Spleen HES staining 10× (left) and 2.5× lens (right). Spleen from PV, PPMF and PTMF mice compared to WT mice are characterized by (*i*) clustered MK, (*ii*) fibrosis and (*iii*) hyperplasia of the myeloid red pulp and blending of the lymphoid white pulp areas (×10 lens). Treatment with Fedratinib induced decreased in MK density and appearance of lymphoid areas in PPMF mice, slightly in PTMF mice and especially in PV mice (×2.5 lens). (**C**) BM silver staining 10× (left) and 2.5× lens (right). No excess fibrosis is shown in WT and PV mice. High-grade BM fibrosis (large deposit of reticulin fibres) and osteosclerosis are shown in vehicle-treated PPMF and PTMF mice. Fedratinib-treated PPMF mice did not display fibrosis and osteosclerosis (*n* = 7) in contrast to vehicle-treated PPMF mice (*n* = 5) and Fedratinib/vehicle-treated PTMF mice, day 57 or 70. (**D**) Spleen silver staining 10× lens. High-grade fibrosis is shown in PV, PPMF and PTMF mice compared to WT mice. Fedratinib treatment did not reduce fibrosis but seemed to worsen it (especially around the blood vessels) in PV and PPMF mice. This effect may be because of shrinking of the spleen in treated animals. Images were obtained (10× or 2.5×/0.3 Zeiss lens) using a Zeiss Axiophot microscope with a Zeiss AxioCam Mrc camera and the AxioVision Rel.4.3 acquisition software (Oberkochen, Germany).

In the PV and PPMF models, histology of the organs with the haematoxylin and eosin/safran (HES) staining reveals that Fedratinib strikingly allowed a return to a quasi-normal architecture of spleen and BM with lymphoid areas (white pulp) appearing in the spleen (Fig.4A and B, Fig. S1A and B). In the PTMF model, no major effect was seen on BM or spleen architecture.

In BM, osteoclerosis was observed in the PPMF and PTMF models during the treatment by vehicle. It was fully abolished with the Fedratinib treatment in the PPMF model but not in the PTMF model (Fig.4A–C).

In the PV model, no fibrosis developed in the BM (Fig.4C). In the PPMF model, no BM fibrosis occurred before treatment, but high-grade fibrosis was observed at end of treatment with vehicle (Fig.4C). Strikingly no BM fibrosis and osteosclerosis were observed after 57 days of Fedratinib treatment (*n* = 6, Fig.4C) showing that Fedratinib treatment prevented the development of BM fibrosis and osteosclerosis in this early or slowly developing MF model. However, no effect on BM fibrosis was observed in the PTMF model. Fedratinib treatment did not reduce spleen fibrosis whatever the model (Fig.4D). At the opposite, an increase in spleen reticulin fibre density, compared to vehicle, was observed after Fedratinib treatment in the PV and PPMF models (Fig.4D). In the PTMF model, no change in the massive network of spleen reticulin fibres was seen in Fedratinib-treated mice.

Overall, Fedratinib contributed to the prevention of developing collagen producing cells or excess of fibre deposition in marrow. However, Fedratinib did not play this curative role in the rapid development of BM fibrosis of the PTMF model or in the treatment of spleen fibrosis in both models showing that JAK2-independent changes within the microenvironment play a crucial role in fibrosis or in the rapid TPO-induced development of fibrosis.

### JAK2^V617F^ allele burden

**Figure 5:**
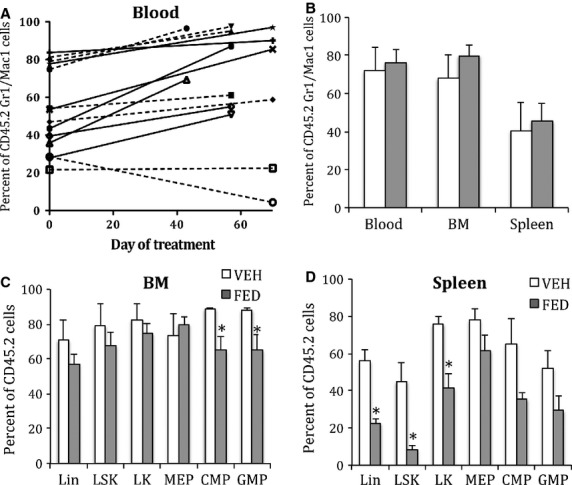
Effect of Fedratinib on JAK2^V617F^ allele burden in the PPMF model. Competitive grafts composed of 2/3 CD45.1 WT and 1/3 CD45.2 JAK2^V617F^ KI BM cells were transplanted into recipient mice. Mean percentage over 33% of CD45.2 cells at start of treatment (22 weeks after BMT) showed proliferative advantage of KI cells over WT cells. (**A**) Percentage of CD45.2^+^ blood myeloid (CD11b^+^/Gr-1^+^) cells (from KI origin) determined by FACS in vehicle- (solid lines) or Fedratinib- (dashed lines) treated recipient mice. (**B**) Percentage of CD45.2^+^ blood, BM and spleen myeloid cells (from KI origin) at end of treatment (*n* = 7) from animals treated by in vehicle (white column) or Fedratinib (grey column). (**C**) Percentage of CD45.2^+^ Lin^−^, LSK, LK, MEP, CMP and GMP cells in BM from animals treated by vehicle (white column) or Fedratinib (grey column), *n* = 4. (**D**) Percentage of CD45.2^+^ Lin^−^, LSK, LK, MEP, CMP and GMP cells in BM from animals treated by vehicle (white column) or Fedratinib (grey column), *n* = 4. Results show that Fedratinib only decreased JAK2^V617F^ allele burden in immature spleen cells. Results are mean values ± SEM, *P ≤ 0.05.

Allele burden was assessed in the PPMF model using Ly5.1 WT recipient and Ly5.1+2 donor competitive mice. Myeloid cells (Mac-1/Gr-1) from JAK2^V617F^ KI donor origin were identified by their Ly5.2 allotype (Fig.1). Allele burden at start of treatment (22 weeks after BMT) in blood cells varied from 20% to 83% and increased with the evolution of the disease (Fig.5A). The treatment had no effect on this evolution compared to vehicle. At the end of treatment, there was no significant difference in the percentage of myeloid cells (Gr1^+^/CD11b^+^) from KI origin assessed in the blood, but also in BM and spleen of Fedratinib- and vehicle-treated mice (Fig.5B). However, focusing on progenitor cells by immuno-labelling, the treatment clearly induced a large decrease (>50%) in ‘allele burden’, *i.e*. the percentages of spleen Lin^−^, LK and especially LSK from KI origin and only the percentages of BM CMP and GMP from KI origin (Fig.5C and D). This result suggests that Fedratinib prevented the mobilization or development of KI stem cells and associated extramedullary haematopoiesis (EMH) in the spleen. This effect may have contributed to the decrease in splenomegaly.

## Discussion

In this study, we investigated the effect of the selective JAK2 inhibitor Fedratinib 10, in mouse models of MPN driven by JAK2^V617F^ or TPO/MPL/JAK2 with increasing disease severity mimicking human PV, human PV with its evolution into PPMF and human ET with a rapid evolution into PTMF. According to the patient scoring system 20, leucocytosis, anaemia and blast counts 18 would classify our PPMF and PTMF models into low- and high-risk PMF groups respectively. In all models, JAK2 inhibition reduced the excess of blood leucocytes, platelets or RBC. Microcytosis was also reduced with decrease in erythroid hyperplasia, when observed. Splenomegaly was also considerably corrected. Interestingly, the effect of Fedratinib varied according to the models with increase in low levels of platelets observed in the PPMF model and decrease in high levels observed in the PTMF model. Similarly, the percentage of granulocytes decreased in the PTMF model but increased in the PV model. Interestingly, development of BM fibrosis and osteosclerosis was strikingly suppressed in the PPMF but not in the PTMF models. Paradoxically, spleen fibrosis was not affected in all models and even appeared worsened. Finally, no decrease in allele burden was observed at the exception of spleen early progenitors, suggesting that they contribute to the decrease in splenomegaly.

Activation of JAK2, through the JAK2, MPL or CALR mutations, has been identified in the vast majority of classical BCR-ABL-negative MPN. In mice, these mutations generate the corresponding MPN phenotype observed in human 1,2 (and personal communication), showing that JAK2 activation is the main pathological contributor towards MPN phenotype. Therefore, the development of JAK2 inhibitors has represented a real hope for the treatment of these diseases. Ruxolitinib, which inhibits JAK2 but also TYK2, JAK1 and JAK3 10 at similar degrees demonstrated durable reduction in splenomegaly and constitutional symptoms with improved quality of life in comparison to placebo-controlled 21 or best available therapy 22 in phase 3 trials and an impact on survival for patients originally randomized to ruxolitinib compared with those originally randomized to placebo in both COMFORT trials 23. Anaemia and thrombopenia were the main limitations and side effects. Ruxolitinib was approved by the FDA for the treatment of intermediate-2 and high-risk myelofibrosis. However, molecular remission including major drop in allele burden has not been demonstrated 21,22,24. It appears as a good palliative drug for patients suffering from MF, but better drugs, including more selective JAK2 inhibitors, are expected to be more effective in treating MPN 25. Fedratinib 11 displays a more JAK2-selective inhibitory activity than ruxolitinib 10. A multi-centre phase I/II trial with high- or intermediate-risk primary or post-PV/ET myelofibrosis demonstrated Fedratinib efficacy in reducing splenomegaly, normalizing WBC counts and significantly decreasing JAK2^V617F^ allele burden for some patients 11. Main adverse events included diarrhoea (10%) and more often thrombocytopenia (24%) and anaemia (35%). Unfortunately, cases consistent with Wernicke's encephalopathy were reported in patients participating in Fedratinib clinical trials and the trials were put on clinical hold as directed by the FDA 26. These events have been attributed to thiamine uptake inhibition 27.

Our MPN murine models were studied in transplanted animals to have homogeneous disease groups. Two models were developed from JAK2^V617F^ KI mice mimicking human PV and evolving into secondary MF (PPMF) 16. The third model resulted from continuous MPL activation by TPO secretion from haematopoietic cells and models MPL^W515L^-positive human PMF or PTMF 18. All models are characterized by splenomegaly with erythroid hyperplasia in the PV/PPMF models and myeloid hyperplasia and anaemia in the PTMF model. Fedratinib was very efficient in reducing erythroid hyperplasia in the PV/PPMF models as shown by the reduced number of MEP, spleen erythroblasts, circulating reticulocytes and RBC, confirming JAK2 inhibition as an option in PV treatment 9. However, it was less efficient in reducing stressed erythropoiesis in the PTMF model and did not correct the anaemia, as we showed for the JAK1/2 inhibitor Momelotinib 28, possibly because of its JAK2 specificity 29. Microcytosis, because of iron deficiency 19, was corrected in all models leading to minor effects on haematocrit and Hb levels. Fedratinib was also very efficient in reducing leucocytosis, but not myeloid hyperplasia in the marrow and spleen. MK hyperplasia was reduced in all models. Regarding platelets, it is not well understood why Fedratinib had the paradoxical effect in reducing thrombocytosis in the PTMF model, as expected from a JAK2 inhibitor, but reducing thrombopenia observed in the PPMF model. Increase number of platelets by a JAK2 inhibitor treatment has been reported in mice 30. As we showed, the mechanism of action may be related to the dual opposite role of MPL inducing either proliferation or blockade of proliferation with low or high levels of stimulation respectively 31. Thus, decrease in MPL/JAK2 stimulation may either increase or decrease platelet counts. Alternatively it may be because of restoration of MPL level and low JAK2 stimulation by a JAK2 inhibitor in JAK2^V617F^ cells 32.

No decrease in JAK2^V617F^ allele burden was observed in the PPMF model in blood, BM and spleen in agreement with reports in ruxolitinib-treated patients 21,22 or mice 33. However, when we focused on progenitor cells, we noticed a decrease in the percentage of LSK from neoplastic origin in the spleen but not in the BM. This result may suggest that JAK2 inhibition by Fedratinib specifically blocks the migration induced by JAK2^V617F^ of early BM cells into extramedullar sites resulting into EMH. Alternatively stem cells residing in the spleen may require a higher JAK2 activation than those residing in the BM possibly because of different stromal environments. These could be the elusive mechanisms resulting into the reduction in splenomegaly in human, a major effect of JAK2 inhibitors on one of the most debilitating symptoms of MF 34. Our preliminary data show that spleen EMH in JAK2^V617F^ KI mice is partially dependent on the CXCR4/CXCL12 axis. The link between JAK2^V617F^ and CXCR4 is not well understood and there are controversial results concerning the level of CXCR4 on CD34^+^ cells and their response to CXCL12 35.

Strikingly, PPMF mice treated with Fedratinib did not develop BM myelofibrosis or osteosclerosis in contrast to PTMF mice. Blockade of myelofibrosis was associated with a decrease in MK hyperplasia, large size and ploidy, suggesting correction of dysmegakaryopoiesis, and decrease in TGF-β1 concentrations in organs that may have contributed to the prevention of fibrosis 36. In contrast, MK hyperplasia/dysplasia was maintained in the PTMF model during the treatment. Overall, this result shows that anti-JAK2 therapy efficiently prevents excessive deposits of extracellular matrix (ECM) proteins and bone formation in low- but not high-risk PMF model and suggests that this effect is mediated through inhibition of MK hyperplasia and dysplasia. Another explanation is that JAK2 inhibition is not as efficient to inhibit TPO-stimulated MK than JAK2^V617F^-stimulated MK and this maybe because of the rapid fibrosis development of this high-risk PMF/PTMF model or different signalling regulation 32,37. In contrast, worsening of spleen fibrosis that could have resulted from the shrinking of the organ was observed in all models. The meaning of this observation regarding human MPN remains elusive because, to our knowledge, spleen fibrosis is not reported in patients. The fact that spleen but not marrow fibrosis persisted strongly suggests that the microenvironment play a role in preventing or resorbing extra fibrous tissues during Fedratinib treatment. Reversion of fibrosis has been reported after BM transplantation and after interferon-α treatment for some patients 38. It has not been reported in large clinical trials with ruxolitinib 22 even if *post hoc* analysis suggested that JAK1/2 inhibition retards the progression of BM fibrosis for some patients 39. It would be interesting to know if this correction was associated to early disease stages. In JAK2^V617F^ overexpressing animal models, reduction in BM reticulin fibre density, without mention about spleen fibrosis or BM osteosclerosis, by several JAK2 inhibitors, including Fedratinib 12, Momelotinib 40, NS-018 41, G6 42 and R723 43 was reported in mice displaying low-grade fibrosis or in early disease stages but not in models displaying high-grade fibrosis. These accumulating data that reinforce our results strongly suggest that correction of BM fibrosis and osteosclerosis development in early MPN phase is strictly mediated through inhibition of JAK2. In contrast, the fact that MK hyperplasia and fibrosis were only slightly modified by Fedratinib in our TPO^HIGH^ models or by other JAK2 inhibitors in JAK2^V617F^ late-phase disease models 43 suggests that the development of advanced stage myelofibrosis and associated pathological megakaryopoiesis become progressively JAK2-independent in MPN and refractory to JAK2 inhibitors. Changes in the microenvironment are capable to induce a MK differentiation independent of the TPO/MPL/JAK2 axis as observed for *c-mpl*^−/−^ mice when treated with 5-FU or by the association of CXCL12 and FGF4. Therefore, other agents acting on MK, the marrow microenvironment such as CXCR4 antagonists or anti-fibrotic drugs seems necessary to treat PMF at advanced stages when JAK2 inhibitors may not be sufficient. Overall, our study shows that JAK2 activation is responsible for deposition of ECM protein by accessory cells and that JAK2 inhibition may prevent this lethal evolution only if applied early in disease evolution.

Overall, this study underlines that JAK2 inhibition, using a JAK2-selective inhibitor, may lead to different effects on fibrosis, osteosclerosis, granulocytosis, erythropoiesis or platelet counts according to MPN disease stage. It gives clues on the mechanisms on how JAK2 inhibition may modulate platelet counts, block marrow fibrosis and osteosclerosis and prevent mobilization of early neoplastic cells in extramedullary sites decreasing splenomegaly. Finally, it predicts that JAK2 inhibitors will be more efficacious with less major toxic effects in early/pre-fibrotic MF patients and low-risk rather than in high- or intermediate-risk PMF patients.

## Supporting Information

**Data S1** Methods. **Figure S1** MKC from WT, PV, PVMF and PMF mice treated by Fedratinib (FED) or vehicle (VEH) are identified in spleen or BM by immunochemistry using a peroxidase-conjugated anti-VWF polyclonal antibody. **Figure S2** TGF-β1 levels in tissue fluids from PVMF mice treated by Fedratinib (FED) or vehicle (VEH).
