# Supplementary material for: JAK2 inhibition has different therapeutic effects according to myeloproliferative neoplasm development in mice
Source: J Cell Mol Med. 2015 Jul 14;19(11):2564–74. doi: 10.1111/jcmm.12608 (PMC4627562; doi:10.1111/jcmm.12608)
Supplement: Supplementary file 1 — Data S1 Methods. Figure S1 MKC from WT, PV, PVMF and PMF mice treated by Fedratinib (FED) or vehicle (VEH) are identified in spleen or BM by immunochemistry using a peroxidase-conjugated anti-VWF polyclonal antibody. Figure S2 TGF-β1 levels in tissue fluids from PVMF mice treated by Fedratinib (FED) or vehicle (VEH). [file jcmm0019-2564-sd1.pdf]

Supplemental Methods:

Flow cytometry (CANTO II®, DIVA6® analyzer, Becton Dickinson, Mountain View, USA) was used to determine cell composition in blood, BM and spleen after RBC lysis and labelling with anti-CD45.1/R-phycoerythrin (PE), anti-CD45.2/allophycocyanin (APC), anti-Mac1 (CD 11b)/ fluorescein isothiocyanate (*FITC*), anti-Gr-1/PerCP-cyanine (Cy) 5.5, anti-B220/APC-Cy7, anti-CD3/Pacific Blue, anti-Ter119/PE-Cy7 and anti-CD71/BV510 antibodies (Pharmingen, San Diego, USA). Appropriated rat IgG2a- or IgG1-conjugated were used as controls. Lineage-negative (Lin-) cells were isolated using APC-conjugated lineage panel (Gr-1, CD11b, B220, CD3 and Ter119). Lin^-, $Sca1^+$, c-Kit^+ (LSK) cells were analyzed from Lin- cells labeled with anti-Sca-I/PE-Cy7 and anti-c-Kit/PerCP-Cy5.5 antibodies. MEP, GMP and CMP progenitors were analyzed with anti-CD34/FITC and anti-FcRγII/III/APC-Cy7. Biotinylated anti-CD45.1/Pacific orange streptavidin and anti-CD45.2/AlexaFluor 700 were used to differentiate between WT (CD45.1+2) and KI $JAK2^{V617F}$ (CD45.2) cells from endogenous WT cells (CD45.1).

For histopathology analysis, spleens were fixed in formaldehyde and sections (4.5 µm) were stained with hematoxylin/eosin, periodic acid Schiff and Giemsa for cytology analysis. Reticulin fibers were revealed by silver staining according to the Gordon Sweet method. Megakaryocytes (MKC) were revealed by histochemistry with a rabbit anti-von Willebrand Factor polyclonal antibody (Dako, Les Ulis, France).

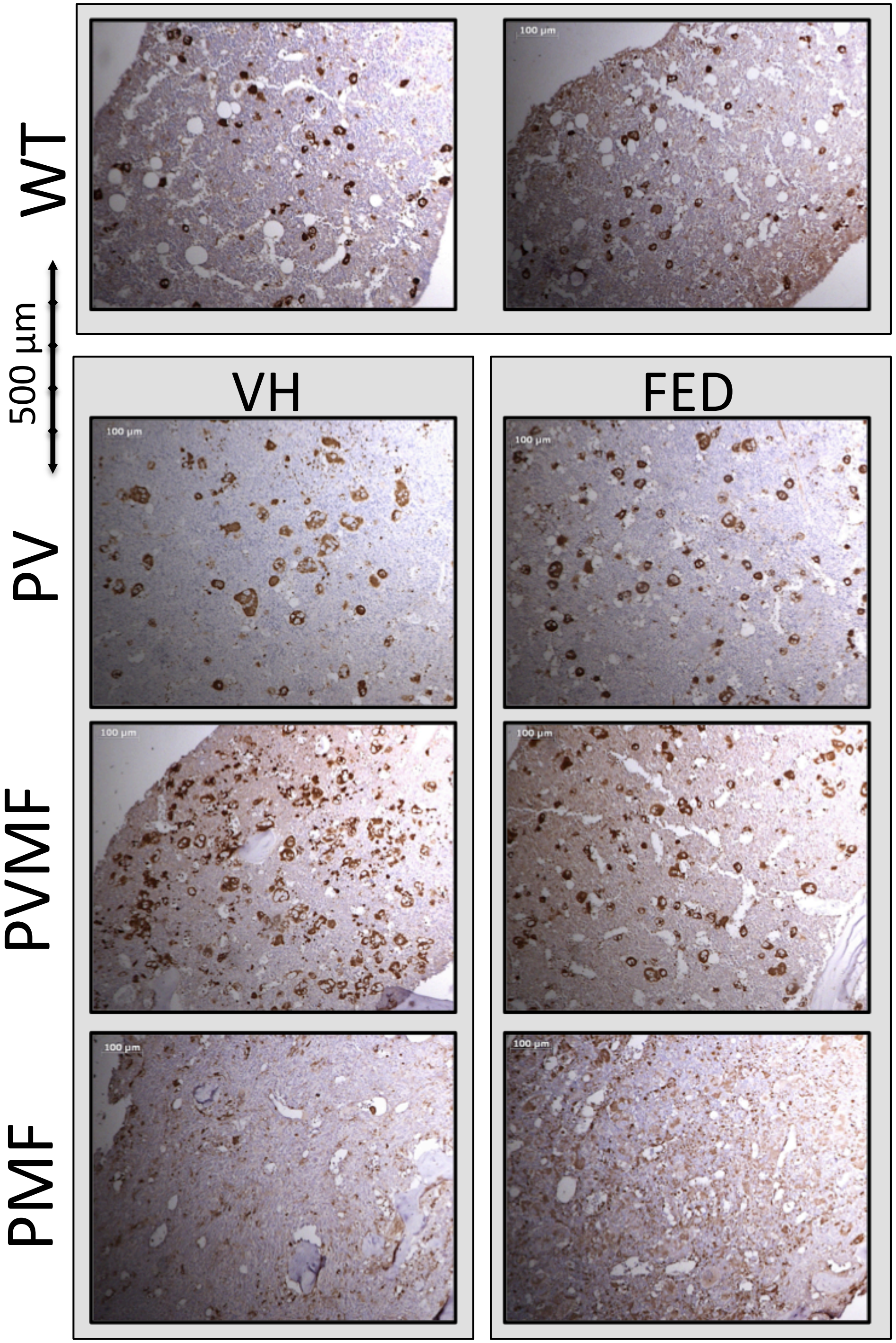

MKC from WT, PV, PVMF and PMF mice treated by Fedratinib (FED) or vehicle (VEH) are identified in spleen or BM by immunochemistry using a peroxidase-conjugated anti-VWF polyclonal antibody. Images were obtained (10X or 2.5X/0.3 Zeiss lens) using a Zeiss Axiophot microscope with a Zeiss AxioCam Mrc camera and the AxioVision Rel.4.3 acquisition software (Oberkochen, Germany).

A BM MKC. Result showed a high MKC density in vehicle-treated PV and especially PVMF mice compared to WT mice and a decrease in BM MKC density in the Fedratinib-treated PVMF mice only. However, MKC size decreased in PV and PVMF mice. BM MKC in the PMF model were pale, dysplatic and necrotic in a hypocellular marrow.

B. Spleen MKC. MKC decreased in density and size in PV and PVMF mice . The lymphoid white pulp areas appeared after Fedratinib treatment in the PV and PVMF mice. MKC in the PMF model were pale, dysplatic and necrotic in a high grade fibrotic spleen.

C. PVMF model. MKC size (in µM) was determined using the microscope gauge. The diameters of 15 (WT) or 25 to 45 (VH and FED) MKC (at lens 16) from a mice treated by Fedratinib (FED) or vehicle (VH) were analyzed. MKC density indicates the number of MKC per field. In spleen, MKC density was decreased by Fedratinib. In BM no variation was observed (not shown). Results are mean value±SEM * $p \leq 0.05$.

Supplemental figure 1,A

Suppl. figure 1,B

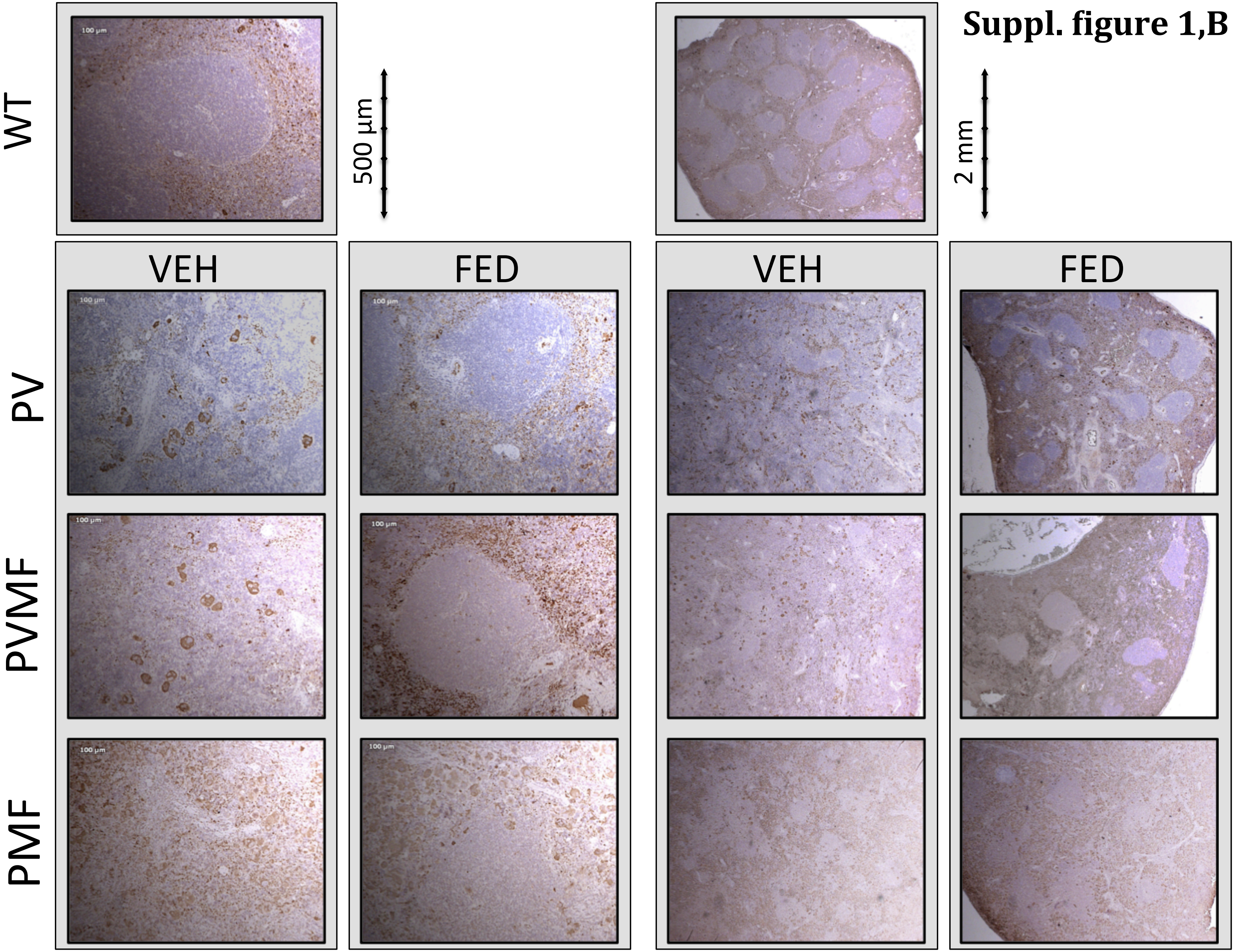

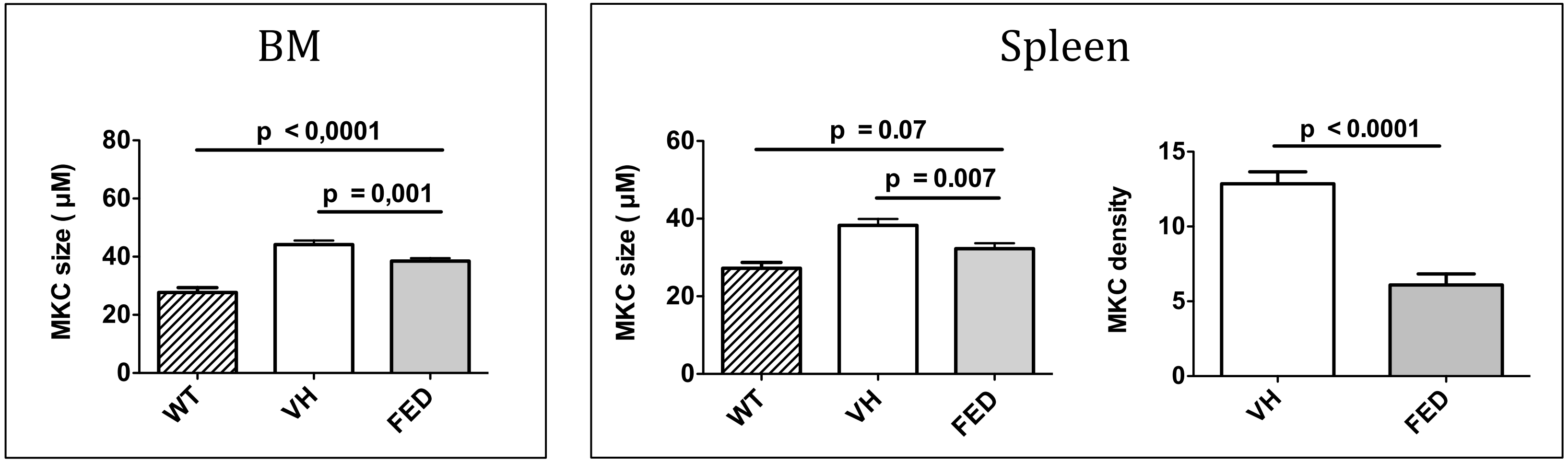

Suppl. figure 1,C

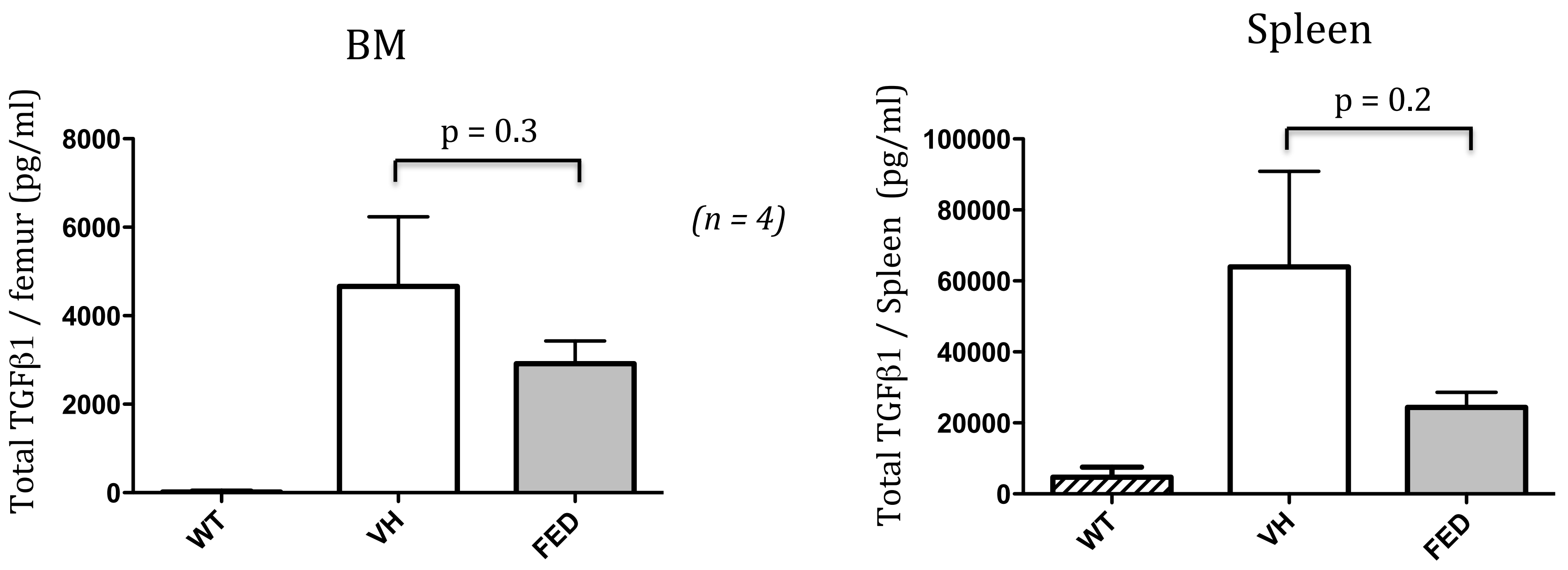

Supplemental figure 2: TGF-β1 levels in tissue fluids from PVMF mice treated by Fedratinib (FED) or vehicle (VEH). TGF-β1 was measured (R&D systems, Minneapolis, MN) in the marrow and part of the spleen extraction fluids. Active forms of TGF-b1 could not be detected (Quantikine Kit, R&D Systems). Decreased of TGF-β1 in Fedratinib-treated could be linked to the decrease in MKC size/density in organs. Results suggest that treatment tended to decrease TGF-β1 concentration in organs that may partially explain fibrosis clearance in BM. However, it does not fully explain the absence in marrow (levels still high) and the persistence in spleen of fibrosis in Fedratinib-treated PVMF mice.

Suppl. Fig. 2
